# Canadian academic nursing librarians: impacts of the COVID-19 pandemic on librarianship practice

**DOI:** 10.29173/jchla29596

**Published:** 2022-08-04

**Authors:** Katherine Miller, Robert Janke

**Affiliations:** 1 MLIS, Reference Librarian, University of British Columbia, Woodward Library, Vancouver, BC; 2 MLIS, Associate Chief Librarian, Research and Administration, University of British Columbia, UBC Okanagan Library, Kelowna, BC

## Abstract

**Objective:**

This study explored changes in the practice of academic nursing librarianship at large Canadian universities during the COVID-19 pandemic with a particular focus on academic nursing librarians’ work with nursing graduate students and nursing faculty.

**Methods:**

Semi-structured interviews were conducted with eleven academic nursing librarians about changes to their librarianship practice during the COVID-19 global pandemic. Interviews were conducted between 20 April and 14 May 2021, discussing experiences during the study period March 2020 to May 2021.

**Results:**

Canadian academic nursing librarians experienced *(i)* the adoption of the completely virtual library *; (ii)* changes to the type and prevalence of online instruction *; (iii)* the discovery that online consultations work well *; (iv)* the discovery of the extent to which relationships are valued and intentional *; (v)* an increase in requests for instruction and co-authorship of knowledge syntheses; *and (vi)* the benefits and challenges of remote work.

**Discussion:**

Experiences were divergent, shaped in part by the institutions’ pre-pandemic practices. Additionally, some participants reported no impact of the pandemic on their research, instruction, and collaborations with nursing graduate students and nursing faculty. In particular, institutions already offering online masters programs in nursing reported less significant disruption. The temporary transition to the completely virtual library revealed benefits of online consultations, opportunities for reaching more students through asynchronous learning, the importance of relationships to nursing liaison work, and value of the flexibility to work remotely .

**Conclusion:**

The COVID-19 global pandemic continues to evolve. With a return to in-person classes at Canadian universities, there is much to learn from the experiences during the first 18 months of the pandemic.

## Introduction

Canadian university campuses and academic libraries closed from March 2020 to August 2021 to all but a small number of faculty, librarians, staff, and students due to the COVID-19 global pandemic. On 11 March 2020, the World Health Organization declared a global pandemic [1] and within a few days, all Canadian universities libraries closed their doors to in-person support [2]. Library staff worked from home, some in-person reference desks transferred online, and subject librarians offered virtual research consultations. This was an unprecedented disruption to all aspects of academic life and society.

Academic libraries support faculty, staff, and students with their teaching, learning and research. One of the groups supported are nursing graduate students. They are working on masters and doctoral assignments, culminating projects, theses, and dissertations, all of which require advanced searching skills. Academic nursing librarians teach information literacy concepts as well as techniques to find, manage, and cite the resources for their studies. Classroom instruction and one-on-one research consultations are some of the ways librarians teach these complex search methods to graduate students [3]. Specifically, as part of their theses and dissertations, nursing graduate students conduct knowledge syntheses and nursing librarians are often involved in teaching the skills for evidence informed practice.

This study investigates how the work of Canadian academic nursing librarians was affected by the pandemic. In particular, it explores how their work collaborating, instructing, and partnering on research with nursing graduate students and nursing faculty may have changed and whether there are any learnings from this pandemic experience that librarians should keep in the post-pandemic academic library.

## Methods

This study received research ethics approval from the University of British Columbia (UBC) Behavioural Research Ethics Board on 13 April 2021. Participants were selected from Canadian Academic Research Libraries (CARL) institutions as their member libraries have doctoral programs and a research mandate [4, 5]. Additionally, only large CARL institutions with total student populations greater than 15,000 and with a doctoral program in nursing were included. Eighteen potential study participants were identified [6]. Eleven academic nursing librarians (61% of the contacted population), consented to participate, and represented diverse geographic areas of Canada including Atlantic Canada, Quebec, Ontario, the Prairies, and Western Canada.

### Interviews and setting

Participant interviews were conducted by the principal investigator. They were one-hour and recorded using Zoom. Participants received the consent form and interview guide as part of the recruitment email. The interviews were conducted using a semi-structured interview methodology. The interview guide was divided into four parts: an introduction, a section on nursing graduate students, a section on nursing faculty and a section of potential additional questions about instruction and research services (e.g., questions about data management). In total there were approximately twenty open-ended questions about experiences of instruction, research support and collaborations with faculty during the pandemic compared to pre-pandemic services and collaborations. Participants were asked to describe what changes they would like to continue post-pandemic.

The interviews occurred over a four-week period from 20 April 2021 to 14 May 2021, during the third wave of the pandemic in Canada [7] During this period, Canadians were starting to receive their first vaccine doses and most Canadian provinces were in strict lockdown as hospitals were nearing capacity [8] and hospital personnel were expressing concerns [9]. Most of the population were still working from home when possible and only essential services were available. Travel restrictions were strictly enforced and were augmented by further restrictions within provinces and regions [10].

### Anonymizing, de-identifying and supplementing interview transcripts

To share the study data, the interview transcripts were anonymized, de-identified and codes (L1 to L18) assigned by a random code generator. Following each interview, the PI saved the Zoom transcript, reviewed it line by line, transcribed verbatim from the video recording, and lightly edited for readability (e.g. removing “um”) to preserve the original intention of the speaker. Personal and identifying information were removed from the transcript text and replaced with square brackets and when possible, using a method described by Saunders et. al. [11] with a generalised description of the content e.g. [more than 5 years]. Next, the participants were emailed the draft transcript and had two weeks to review it to make corrections [11]. Participants were able to redact content that could identify them, and also to make additions to the transcript to clarify their answers. The practice of sharing the transcripts with the interviewees is used to confirm the researcher’s understanding and to collaborate on the construction of the interview transcript with the participant [12]. Collaborative interview transcript methodology is adapted from the narrative inquiry method [12].

As the study population is very specific, to reduce the possibility that participants can still be identifiable by their responses, unique aspects of the nursing librarians’ work (e.g. their research interests) were redacted from the transcripts. The institutions’ names, cities, regions, unique graduate programs, unique course offerings or institution specific learning objects, learning management systems, and software were generalized e.g. [another software] [13]. Additionally, unique features of the participants’ home life such as children, workspace, internet connectivity were redacted from the transcript to preserve the participant’s anonymity. One of the limitations of the study is the transcripts are not exact records of the interviews and some of the meaning has been lost due to the anonymization. Additionally, the redaction of personal information about the participant’s remote work situation is a limitation to this study’s scope of findings.

## Coding

After finalizing the transcripts with the participants, the PI used a qualitative content analysis approach to code the data [14] in NVivo 12. She used an open coding approach and coding was reviewed by the second researcher. Coding was accomplished through a comprehensive and iterative approach to identify topics raised by the participants and language used to describe their experiences. Ninety-three codes were applied to the transcripts. Using a method similar to that discussed by Ji Young Cho and Eun-Hee Lee [15], from these codes, themes were identified, revised inductively and used to further classify themes into key findings.

## Results

Analysis of the study data revealed a wide range of themes and a heterogeneity of experiences for Canadian academic nursing librarians. In this study we focus on six themes which emerged as the most common changes to research, instruction, and collaborations with nursing graduate students and nursing faculty since the COVID-19 global pandemic. During the pandemic, Canadian academic nursing librarians experienced *: (i)* The adoption of the completely virtual library *; (ii)* changes to the type and prevalence of online instruction; *(iii)* the realization that online consultations work well *; (iv)* the discovery of the extent to which relationships are valued and intentional *; (v)* the increase in requests for co-authorship and instruction supporting knowledge syntheses; and *(vi)* the impacts of remote work (Table 1).

**Table 1:** Six themes

Themes	Findings	# of times theme mentioned across all interviews	How many participants mentioned it (N=11)
**(1)Adoption of the completely virtual library**	Change from in-person to online services	25	11
	Mode of delivery is online	78	11
	Total:	103	11
**(2)Changes to the type and prevalence of online instruction**	New or updated asynchronous content	7	6
	Recording of library class (without students being present)	7	3
	Only asynchronous –lost synchronous class	7	3
	Total:	21	10
**(3)Online consultations are perceived to be an improvement**	More convenient	8	7
	Pre-work for consults	3	3
	Reach new students	1	1
	Work well in general	6	3
	Recording consult	1	1
	Screensharing Patron shares their screen	9 4	7 4
	Zoom easier to connect	5	4
	Total:	37	11
**(4)Relationships are valued and intentional**	Importance of relationships	18	7
	Fewer opportunities to connect	14	8
	Total	32	10
**(5)Increase in requests for instruction/consultations and co-authorship of Knowledge Syntheses**	Nursing graduate students	8	4
	Nursing faculty	8	3
	Total	16	5
**(6)Impacts of remote work**	Work-life boundaries challenging	4	3
	Workload overwhelming	3	2
	Total –challenges	7	3
	Librarian experiences flexibility in workday	1	1
	Librarian experiences more time	6	4
	Fewer distractions	1	1
	Total –benefits	8	6

### The completely virtual library

The most significant change during the pandemic was the rapid shift to online content and services. The majority of participants reported their research and instruction consultations that had previously been in-person transitioned quickly to online only. Library services and collections were provided online and accessed remotely. For many their instruction and consultations pre-pandemic had been a very in-person model. One of the interviewees summarized the changes noting the “biggest cultural shift” was to the completely virtual library [16].

### Changes to the type and prevalence of online instruction

All eleven participants discussed changes to their library instruction during the study period. First, the transition from in-person to online instruction was a substantial change for some of the participants. At some institutions, the transition to online instruction was a significant shift in practice [17-22]. Second, the shift to online instruction included the creation or adaptation of asynchronous learning objects. Third, the shift from in-person to online synchronous instruction resulted in a substantial increase in work to transition their class to the new online format. There was a significant impact on their workload to ensure that it was active and engaging [23]. Fourth, for some, asynchronous content replaced their synchronous library instruction. New content was created to replace pre-pandemic in-person orientations and classes [13, 17, 24]. Three of the participants [16, 17, 24] reported the loss of the synchronous library instruction to their graduate nursing students. For one respondent, they no longer met with their nursing graduate students all together in a synchronous online setting. They explained, “my synchronous teaching got cut pretty much almost entirely from nursing because of the way that they chose to do their curricular changes for COVID” [16]. Others reported this change was of benefit to the nursing graduate students to be able to view the recording at their point-of-need [24]. For some, this new asynchronous content expanded their reach to graduate students who had not received librarian-led instruction prior to the pandemic [16, 22].

### Research consultations offered online are perceived to be an improvement over in-person consultations

There were a number of observations about how librarians' research consultations with graduate students have changed during the pandemic. With the closure of campuses and libraries during the pandemic, all research consultations transitioned to online-only. All of the participants reflected on advantages of conducting their research consultations online over the former in-person consultations. First, the ease of access for the graduate students from anywhere in the world (their home, a clinical placement, etc.) without needing to come into the physical library, was an important benefit of online consultations [16, 18-21, 23, 24]. Several participants [13, 16, 21, 22] noted the widespread availability of Zoom software, and its ease of use to connect with students. Furthermore, most of the participants identified the ability to screenshare as an improvement [13, 17, 19, 21, 23, 25]. In particular, they mentioned the students’ ability to share their screens as an improvement in online consultations [13, 21, 23, 25]. Online consultations and instruction require computer equipment and internet connectivity and one of the participants noted that poor connectivity sometimes resulted in unsuccessful screensharing [22].

Several participants explored ways to create efficiencies and improvements in their online consultations over their former in-person research consultations. A couple of them mentioned they conducted a more thorough reference interview than they had pre-pandemic [23]. They asked more questions and prompted the student to reflect on their own research needs. One created new materials to support the students’ documentation of their search strategies including databases searched [20]. Another participant mentioned that since they had changed during the pandemic to recording their instruction, the students reviewed their library instruction recording prior to meeting with them [24]. Similarly, another participant requested their students complete online modules [19], prior to meeting with them. The capacity to record the online consultation was another advantage of the online consultation [19] as the student did not need to take notes and could focus on the conversation and refer back to the recording, if needed. The participants reported more in-depth conversations [23] and improved quality of interaction in the consultation [16] One participant explained that they do not plan to return to offering in-person meetings for students or faculty post-pandemic [16].

During the pandemic, some of the academic nursing librarians’ experienced increases in the number of research consultations with nursing graduate students [20, 22-25]. However, others reported that support of nursing graduates and nursing faculty decreased [16, 18, 19]. Clearly, there was not a consistent experience of the impact of the pandemic in this area. It is unclear to the authors why this differed between institutions as there was not a consistent factor that came out in our data pointing to cause of the increase or decrease in consultations during the first year of the pandemic.

### Discovery of the extent to which relationships are valued and intentional

Most of the nursing librarians spoke about the strength of their relationships pre-pandemic with nursing faculty and nursing graduate students [13, 16, 19, 21-24]. They identified how important these relationships were to their work to be aware of the needs of students and faculty, and to provide responsive support. Several of the participants mentioned that during the pandemic, being off campus and meeting students and faculty only online did not give them the same opportunity to build these relationships. Pre-pandemic, many of the nursing librarians noted that they would see students and faculty on-campus and have opportunities to stay in touch about their research and answer questions. However, some still found ways to foster these relationships through participation in online retirement parties, attending virtual meetings, and spending time at the beginning or end of online meetings to connect and catch up [21, 22].

These interviews also showed challenges in relationships for new librarians. Librarians who had been in their positions for less than 5 years, did not have the same networks to rely on and mentioned that remote work led to lack of awareness of some tools available [20] and a desire to engage more with library colleagues [25]. They expressed how difficult it was to not work in-person with library colleagues, to not be able to ask a colleague for a second opinion or to learn about institution specific resources available to them as researchers themselves.

### Increase in requests for co-authorship and instruction supporting knowledge syntheses

At the beginning of the pandemic, other than COVID-19-related research, most research at Canadian universities was suspended [26]. This affected the academic nursing librarians in a number of ways. First, some nursing faculty redirected their research to knowledge syntheses [17] and collaborated with academic nursing librarians as co-authors on these reviews [21]. All eleven of the academic nursing librarians reported that they co-authored knowledge syntheses during the pandemic. The number performed during a year was the nursing librarian’s choice – with time constraints and workload being mentioned as some of the criteria used for making this choice [16, 18, 20, 22]. Five of the eleven academic nursing librarians [16, 17, 20, 21, 23] mentioned they observed an increase in knowledge syntheses during the pandemic. Second, nursing graduate students were unable to continue practicums and other clinical experiences as hospitals prioritized caring for the most vulnerable patients and those infected with COVID-19. Several nursing librarians reported that some instructors chose to assign major papers with a comprehensive searching component or full knowledge syntheses to their graduate students [16, 18, 20]. COVID-19 related subjects were the focus of some of the knowledge syntheses conducted by nursing graduate students and faculty [16-18, 22].

### The impacts of remote work

Many of the interviewees noted that working remotely from home was a substantial change to librarianship practice. Prior to the pandemic, academic librarians rarely worked from home as the needs of in-person reference and research consultation, in addition to instruction and work with physical collections, required working in the physical library. Due to the pandemic, there was a rapid transition from in-person campus activities to working from home and working online only. Within one week all work that had formerly been in-person transitioned to online.

This change to working from home had several impacts. Some of the positive changes were flexibility in the work schedule and additional time which some used for new research projects or outreach. One respondent identified that due to the flexibility in the work schedule, they no longer felt bound to the 9 to 5 schedule and found that freedom better matched the needs of their work [24]. Others reflected that they had more time to work on their research and projects because they were working from home. They explained that they gained the time they would usually spend traveling from home to work and back each day [21] as well as not needing to commute on campus to in-person meetings and teaching [16, 25]. Another reported they were able to increase outreach to faculty by attending their faculty council meetings [22]. Remote work was identified as an option that some of the nursing librarians would appreciate post pandemic [19].

Working from home presented challenges to some of the participants. Competing needs such as family at home was sometimes distracting [17]. Additionally, others mentioned the blurring of work and home life lead to overwork for some participants [13, 23].

## Discussion

This study found that the COVID-19 global pandemic resulted in Canadian academic nursing librarians’ research, instruction, and collaborations with nursing graduate students and nursing faculty shifting to a completely virtual environment, which was a substantial change to practice. Starting in March 2020 with the closure of university campuses and the move of all Canadian post-secondary instruction online, nursing faculty across the country quickly needed to adapt their instruction to the online environment [27]. As nursing faculty moved their instruction online, academic nursing librarians reported a need to create, adapt, or update their online asynchronous and synchronous learning materials for nursing graduate students.

This transition seems to have been less challenging for some academic nursing librarians than for others. Several of the academic nursing librarians reported that there was little change to their practice due to the pandemic [13, 17]. Over half of the participants explained that the masters level nursing programs at their institutions were already either partially or completely online prior to the pandemic [13, 16, 18, 19, 22, 24], and as a result they had already been conducting some library instruction and research consultations for their masters students online. One participant observed, “Some of our programs, […] already had a big online component anyway, so I think we're also better prepared than a lot of our colleagues to do that [transition online at the beginning of the pandemic]” [24]. As Stevens, Hinton and Brown [28] reported, academic nursing librarians reported a high level of comfort providing reference, research, and instruction to nurse student graduates online. Even so, the widescale adoption and ease of use of tools such as Zoom was felt by some participants to be a notable change to practice [13, 22]. Others identified their existing embeddedness in the online instruction for their nursing departments as key to their ease of transition during the global pandemic [19].

Librarians have offered virtual reference services and online instruction for decades. These have been offered as secondary services, supplementing the core in-person services. The shutdown of physical academic libraries completely for research consultations and instruction for graduate students is a shift on a scale that has not been experienced in such a universal way pre-pandemic.

### Research consultations and instructions in the completely virtual library

Academic nursing librarians have offered some types of online instruction such as online tutorials, videos and recordings of screencasts for decades. The shifts observed in this study are in the scale and pervasiveness of online instruction. Additionally, while online reference has been offered at many institutions pre-pandemic, during the pandemic it was the only option and offered extensively by all the participants. Several of them described the online consultations as an improvement over their in-person consultations. Improvements that were highlighted included screen sharing, convenience for students to obtain services wherever they were, and the option to record the consultations so a student can refer to it later if needed.

The increase in asynchronous content was highlighted as an improvement as it reached a greater number of students, and is potentially reusable [20, 22]. However, some concerns were raised by one participant that nursing graduate students who had synchronous instruction pre-pandemic now were supported less during the pandemic as they only had access to the asynchronous materials and lost their synchronous class. There were concerns the nursing faculty may not invite the nursing librarians to offer synchronous instruction post-pandemic [16]. Additionally, as has been reported elsewhere [29], the impact of transitioning lesson plans from an in-person setting to an online one, increased the librarians’ workload.

Several of the participants referred to asynchronous learning materials they had created pre-pandemic. While some created new asynchronous materials or revised older materials, they noted this was something they were already planning pre-pandemic and would have happened regardless of the pandemic [17]. And some participants refreshed content that they already had online [18]. Some of the nursing librarians referred to existing online asynchronous content that they had created prior to the pandemic and its value, especially during the pandemic and the need for entirely online services [19]. While several of the interviewees mentioned they had explored ways of to improve their online consultations during the pandemic, it is important to acknowledge that academic librarians are continuously exploring and improving services, and this is not in itself due to the pandemic. Additionally, some techniques that were newly employed by some academic nursing librarians due to the pandemic were already in use at other institutions prior to the pandemic. While these practices may not be new for many academic nursing librarians, there was clearly experimentation on how to make them more efficient and effective for researchers. The academic nursing librarians responded to researchers' changing needs during the pandemic with reflection and innovation of their core research consultation services.

### Knowledge syntheses a central part of nursing librarians’ work

Health sciences librarians, including academic nursing librarians, are important members of research teams for knowledge syntheses [30]. Academic nursing librarians contribute as both authors and instructors. All eleven participants reported that they collaborated on knowledge syntheses as co-authors and provided instruction to graduate students. While these are not new roles, they were highlighted as central roles particularly at the beginning of the pandemic as many faculty and graduate student research projects were paused [17]. These may have been key reasons some of the nursing librarians saw increases in both consultations and requests for support with knowledge syntheses [16, 17, 20, 21].

### Nursing librarians would like the option to work remotely

Academic librarians often work in shared or open offices, and the ability to have quiet space to work is a benefit that some found working from home. The participants’ home environment, including space to work, family members and other factors, resulted in varying work environment experiences for nursing librarians during the pandemic. Although this study did not ask about the participants’ workspaces and details of their working from home, there were some observations. For some it was very positive, for example, one participant reflected, “I’d love for it to stay all remote. Ideally, I’d like to go back to the office when I need to or when I want to. But I think like, speaking about my work, any meeting that I could attend remotely, I probably would” [16]. Depending on their personal circumstances, for some of the participants, working from home meant having fewer interruptions and a quiet space for concentration on projects and research. Several of the interviewees mentioned they would appreciate the option to work from home some of the time [21, 22].

The findings from this study offer suggestions for library support of nursing faculty and graduate students’ research and teaching, and implications for academic nursing librarians’ practice [14]. The following experiences offer potential learnings for post-pandemic academic libraries.

Two of the interviewees (L1, L15) highlighted how the services created in response to the global pandemic have inspired innovations. In response to the pandemic, there are now better services for students and researchers at a distance, as this was the shared experience of all students and researchers in Canada from March 2020 to September 2021. L15 highlighted the advantage now for students on placement or practicum:

I am so pleased at being better able to support our students who are on placement. Before, it was just sort of haphazard and some of these students were in northern rural and remote communities, and so if a book was in print, it was just like ‘Oh well too bad, you can't have that.’ And we've started to get creative about, oh wait, we could ship it to them, or we can scan this, or we can do a Zoom meeting and they don't have to be on campus. And all these little things, I think has really improved our service for those people who are always kind of marginalized, I think on campus because of how they're treated. We've had, as I said, we've had students in remote communities where they just can't get to a physical library and they just aren't going to be able to. So, that's been really big [24].

### Limitations of this study

A limitation of this study is that we did not ask the participants details about working from home. For example, challenges with dedicated workspace (e.g., equipment, ergonomics, internet connectivity, or the complexities of families all being in lockdown and working from home). Details of personal life obligations such as children or other dependents were not included in the data.

## Conclusion

Academic libraries in Canada reopened all physical branches to in-person services in September 2021. While the COVID-19 global pandemic disrupted all aspects of life, including higher education, academic nursing librarians adapted perhaps more easily than some of their colleagues to the changes brought on by the pandemic. For some of them, their graduate nursing programs had already moved online and some research and instruction were already offered online as well. The global pandemic shifted all academic work online and as a result transitioned academic libraries to a completely virtual model during this time period. With the return to in-person services, the learnings of the first 18 months of the pandemic will serve as some guideposts of what to keep from this experience. As one participant expressed so poignantly, the COVID-19 pandemic has given all who work with nursing researchers an even deeper respect for them.

One of the things I think that I’ve appreciated about the pandemic and working with nursing students and faculty is a greater awareness of what's at stake for them. You know, we didn't really think much about them getting sick on the job, and now I do, and I can see the stress that that puts them under. And thinking about the moral distress they feel when they're working and they need time off, and again this was something that was kind of percolating in the back of my mind, but the pandemic really has made it real. And so, I have, I think, a new way of understanding that it's about the relationships are important, that it's critical to build those relationships over time, and that trust is important, trusting your colleagues, trusting the whole system. And we see now what happens when it doesn't work and when people don't trust the system or lose that faith in the way things are supposed to be. So, I’m hoping that I have a little more compassion for people who are struggling in this job and yeah take more time to build those relationships [24].

The authors hope that these innovative instruction and research services for nursing graduate students and nursing faculty may help identify practices that could be adapted to other contexts in order to build better library services post-pandemic. Canadian academic libraries have adapted to the challenges of the COVID-19 global pandemic and provided more accessible services to their users and more flexibility for academic nursing librarians. These examples offer models of what is possible as we envision post-pandemic library services and many areas for further research.

## Data Set Availability

The research ethics application and certificate, transcripts, consent form, interview guide, codebook and NVIVO file with coded transcripts are available in UBC Dataverse 10.5683/SP2/8RFCMB

## About the authors

The authors of this study are two nursing librarians at UBC Library. The University of British Columbia (UBC) has a very large student population with approximately 66,000 students in total with 11,000 graduate students distributed between two campuses in Kelowna and Vancouver, British Columbia. The UBC Library opened in Vancouver, BC in 1914 and in 2005, when the Okanagan University College became UBC Okanagan, the library systems also merged. Some aspects of the UBC Library continue to mirror the organizational structures of the campus structures they support. At UBC Okanagan, the School of Nursing is situated in the Faculty of Health and Social Development, whereas at UBC Vancouver, the School of Nursing is within the Faculty of Applied Science. The nursing programs and faculty are also independent. UBC Library staffing matches those structures with a nursing librarian at each campus. In January 2021, the authors both started six-month study leaves and had the opportunity to conduct this research.
